# Selenium-binding protein 1 as a tumor suppressor and a prognostic indicator of clinical outcome

**DOI:** 10.1186/2050-7771-1-15

**Published:** 2013-03-01

**Authors:** Wancai Yang, Alan M Diamond

**Affiliations:** 1 Department of Pathology, School of Basic Medical Sciences, Xinxiang Medical University, 601 East Jinsui Dadao, Xinxiang, 453003, China; 2 Department of Pathology, University of Illinois at Chicago, Chicago, Illinois, 60612, USA

**Keywords:** Selenium, Selenium-binding protein 1, Glutathione peroxidase 1, Cancer, Biomarker

## Abstract

Selenium is a trace element that plays a critical role in physiological processes and cancer prevention, whose functions may be through its effects on selenium-containing proteins. Selenium-binding protein 1 (SBP1) is a member of an unusual class of selenium-containing proteins that may function as a tumor suppressor in multiple cancer types and whose levels have been shown to be lower in cancers as compared to corresponding normal tissues. This review is intended to summarize recent advances in gaining an understanding of the significance of SBP1 in carcinogenesis, and suggest that SBP1 could be developed as a potential biomarker for cancer progression and prognosis.

## Introduction

Selenium is an essential trace element whose consumption has significant impact on a wide variety of health outcomes. For example, Keshan disease, a congestive cardiomyopathy, is associated with low concentrations of selenium in the soil in the geographical area of Keshan County in China [1]. Higher intake of selenium is associated with a reduced incidence or severity of a wide range of human disorders, including muscle and cardiovascular disease, cancer, endocrine dysfunction and neurological disorders [2]. In particular, geographic, animal and human epidemiological studies, have shown that lower concentrations of selenium in serum is associated with increased risk of cancer and that selenium supplementation may, in some cases, reduce the risk of several cancer types, including those of the esophagus, colon, prostate and liver [3]. The mechanisms of cancer prevention remain unknown but might be linked to enhancement of anti-oxidant defenses, increasing the immune response and being anti-inflammatory.

Selenium is a constituent of proteins of several classes. Selenium-containing proteins generally fall into three categories [4,5]. One of these classes includes those proteins containing selenomethionine in which selenium, due to its structural similarity to sulfur, incorporates non-specifically into the sulfur-containing amino acid [6]. The most common and best studied class of selenium-containing proteins includes those that contain the amino acid selenocysteine (Sec). Throughout evolution, from bacteria to humans, Sec is incorporated co-translationally by its insertion in response to UGA codons that otherwise would serve at translational termination signals. In eukaryotes, the recognition of UGA as Sec requires a regulatory sequence, called a SECIS element, in the 3’-untranslated region of the selenoprotein mRNA [7]. All in-frame UGA codons contained in a SECIS-including mRNA are recognized as encoding Sec, which is incorporated into the elongating peptide by a unique translational machinery that includes a highly unusual tRNA, an elongation factor as well as other proteins required for selenoprotein synthesis [2,8]. There are 25 human Sec-containing selenoprotein genes and 24 in the mouse [5]. The best studied Sec-containing protein is the ubiquitously expressed glutathione peroxidase-1 (GPx1). This enzyme uses reducing equivalents from glutathione to detoxify lipid and hydrogen peroxides and its levels are sensitive to selenium availability. Changes in the levels of GPx1 have been associated with a variety of human diseases. The loss of one GPx1 allele has been frequently observed during the development of several common cancer types, including those of the breast, lung and colon as well as those of the head and neck [9]. An additional class of selenium-binding proteins includes those proteins in which selenium is bound to the peptide, but not incorporated into an amino acid as Sec. One such protein is the selenium-binding protein 1 (SBP1) [10].

### Identification and biological functions of SBP1

The human selenium-binding protein gene (SBP1, SELENBP1 or hSP56) was first cloned in 1997 [11] and has been suggested to mediate the intracellular transport of selenium [11,12], although evidence for this role has not yet been provided. *SBP1* is located on chromosome 1 at q21–22, and is the homologue of the mouse *SP56* gene that was originally reported as a 56 kDa mouse protein that stably bound ^75^selenium [10]. The human cDNA contains a 472 amino acid encoding open reading frame [11]. Chen et al. has detected two 56 kD SBP1 isoforms in human lung cancers using Tandem mass spectrometry and 2-D western blot analysis, in which an acidic isoform (457AA) was observed at lower levels in lung adenocarcinomas compared with normal lung, with two additional, more acidic SBP1 isoforms only observed in normal lung tissue [13].

The SBP1 protein has been detected in both the nucleus and cytoplasm, as assayed by immunohistochemical staining [13,14] and it is expressed in a variety of tissue types, including the heart, lung,kidney and tissues of the digestive tract. *SBP1* is well conserved in eukaryotic evolution, with a BLAST search revealing a better than 65% identity between the human gene and that of organisms as diverse fish, birds, urchins and *Chlamys farreri*, a bivalve (unpublished observation). The form of selenium in SBP1 is unknown as is the nature of its association: the selenium remains bound to the protein when electrophoresed in SDS acrylamine gels but dissociates at extremes of pH [10].

The function of SBP1 is unknown although it may be involved in intra-golgi transport [12]. Recently, SBP1 has been shown to be a target of the hypoxia-inducible factor-1 alpha (HIF1α) [15] and to directly interact with von Hippel-Lindau protein (pVHL) which may play a role in the proteasomal degradation pathway in a selenium dependent manner [16]. SBP1 has also been proposed to serve as a marker in colonic cell differentiation [14].

### Lower SBP1 levels in cancer are associated with poor clinical cancer prognosis

Interest in SBP1 as a prognostic indicator was stimulated in 2004 when gene expression profiling for predictors of outcome in the management of pleural mesothelioma indicated that *SBP1* was among a group of 27 genes whose expression segregated “good risk” from “bad risk” post-surgery in mesothelioma patients [17]. Subsequently, significantly reduced levels of SBP1 in tumors as compared to the corresponding normal tissue has now been documented in cancers of the ovary, lung, esophagus, colon, stomach, liver and in uterine leiomyoma [13,14,18-27]. Reduced expression of SBP1 is also associated with tumor progression. SBP1 levels are reduced in Barrett’s esophagus (BE), BE with low-grade dysplasia, BE with high-grade dysplasia, and are significantly reduced in esophageal adenocarcinoma [21]. Similarly, lower levels of SBP1 are gradually reduced with gastric cancer progression [24]. In lung adenocarcinoma, SBP1 protein isoforms and mRNA levels were significantly decreased in poorly differentiated adenocarcinoma compared to the moderately- and well-differentiated adenocarcinomas [13]. Lower levels of SBP1 were also associated with the late stages of colorectal [14,19] and lung cancer (T2-T4 versus T1) [13].

Several clinical studies have provided evidence indicating that lower SBP1 levels are associated with worse prognosis of the cancers of lung, colorectal, stomach, and liver [13,14,19,22,23,26,27]. Our studies have demonstrated an association between SBP1 expression, as assayed by tissue microarray (TMA), with disease-free survival as well as overall survival among stage III colorectal cancer patients using Kaplan–Meier analysis [14]. The conclusions drawn in this study have been supported by the work of others. Chen et al. reported that low levels of the native form SBP1 protein (460aa) was significantly correlated with poor survival among patients with lung cancer [13]. More recently, Huang et al. presented data that SBP1 was an independent risk factor for overall survival and disease recurrence; patients with lower SBP1 expression as determined using tumor microarrays experienced shorter overall survival periods and higher rates of disease recurrence [26]. While the expression or alterations of SBP1 in gastrointestinal stromal tumors (GISTs) remains unknown although many advances and management have been made [28,29].

### Reduced levels of SBP1 are associated with epigenetic changes

We have previously reported that SBP1 protein and mRNA expression were dramatically lower in human colorectal cancer [14] and recent studies have indicated an association between epigenetic changes of *SBP1* and its reduced expression. Our group has provided data indicating that reduced SBP1 expression was linked to the hypermethylation in the SBP1 promoter using methylation analysis; SBP1 promoter methylation in cancer tissues was much higher than those in the adjacent normal mucosa [20]. These data clearly demonstrate that *SBP1* promoter methylation may be one of the mechanisms responsible for SBP1 downregulation in human colon cancers. Similar results were observed in human colorectal cancer cell lines including HCT116, SW620, SW480, HT29 and Caco2 cells in which lower or undetectable SBP1 levels were strongly associated with *SBP1* promoter hypermethylation. In addition, *SBP1* promoter hypermethylation led to reduced *SBP1* promoter transcriptional activity *in vitro*[20]. Treatment with 5’-Aza-2’-Deoxycytidine (Decitabine, DAC) reversed *SBP1* promoter methylation and stimulated its expression in human colorectal cancer cells [20].

Epigenetic changes in *SBP1* were also observed during esophageal carcinogenesis where the proximal regions of the *SBP1* promoter was hypermethylated during the progression of Barrett's esophagus to adenocarcinoma as well as in esophageal cancer cell lines [21]. In addition, posttranscriptional mechanisms, such as the appearance of isoforms resulting from differential splicing, seemed to modulate SBP1 protein levels, as did single nucleotide polymorphisms (SNPs) [21]. As in the case of colon-derived cell lines, the inhibition of methylation could restore partial expression of SBP1 in esophageal cancer cell lines [21]. In contrast, the treatment of A549 lung adenocarcinoma cells with the methylation inhibitor 5-azacytidine did not affect SBP1 expression [13].

### *SBP1* as a putative tumor suppressor

Several lines of evidences have provided support for a tumor suppressor role of *SBP1*. Analysis of tumor proliferation status using the Ki-67 proliferation marker indicated that down-regulated expression of SBP1 resulted in increased cell proliferation and decreased differentiation in lung adenocarcinomas [13]. Inhibition of SBP1 by siRNA effectively increased cell motility, promoted cell proliferation, and inhibited apoptosis in hepatacellular carcinoma cell lines [26]. Conversely, ectopic SBP1 expression in colorectal cancer cells resulted in inhibition of cancer cell proliferation and the induction of apoptosis, attenuated cancer cell migration *in vitro*, and significantly inhibited cancer cell growth in nude mice [20]. These findings clearly demonstrated a likely tumor suppressor function of SBP1, although determining the underlying molecular mechanisms requires further investigation.

### Inverse regulation and interaction between SBP1 and GPx1

We recently reported that ectopic expression of SBP1 was sufficient to cause an approximately 50% reduction in the activity of the GPx-1 selenoprotein in human HCT116 colon cancer cells without affecting GPx1 mRNA and protein levels [30]. However, increased expression of GPx1, achieved either by transfection of a GPx1 expression construct or by supplementing the media with selenium, resulted in the significant reduction of SBP1 protein and mRNA levels. Selenium titration studies, either in the presence or absence of GPx1, established that the opposing regulation of these proteins was not due to a simple competition for available selenium, and this conclusion was supported by co-immunoprecipitation studies that indicated that SBP1 and GPx1 formed a physical interaction. These results were extended to mice which indicated that increasing GPx1 levels by dietary selenium supplementation resulted in a decline in SBP1 levels in colonic and duodenal epithelial cells [30]. These studies were extended by the work of others in which it was shown that SBP1 and GPX1 formed nuclear bodies and also co-localized under oxidative stress, and that decreased SBP1 was associated with increased GPX1 activity and correlated with vascular invasion in freshly isolated clinical hepatocellular cancer tissues [26]. These results were similar to our most recent data indicating that there is an inverse association between SBP1 and GPx1 levels in human colon (Yang et al., not published) and prostate, as well as the demonstration of an association between higher GPx1 levels and clinical Gleason grade of prostate cancer tissues [31]. These data have collectively demonstrated both an inverse regulation and physical interaction between the representatives of two distinct classes of selenoproteins, each implicated in cancer etiology.

## Conclusion

**Figure 1:**
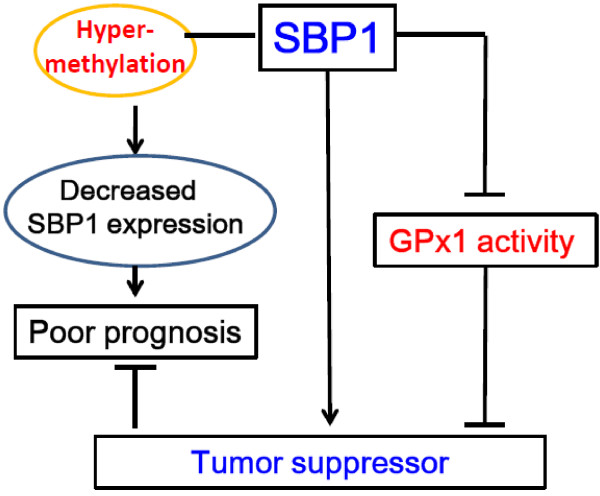
Proposed roles of SBP1 in carcinogenesis and prognosis.

SBP1 is expressed in normal tissues, but its levels are reduced in multiple types of cancer. Reduced levels of SBP1 likely result from both epigenetic and posttranscriptional alterations. SBP1 is inversely regulated and interacts with GPx1, and these effects might be mediated, at least in part, by selenium. Clinically, the reduced expression SBP1 is linked to poor survival of cancer patients (Figure 1). Therefore, SBP1 SBP1 expression may be developed as an indicator for the monitoring of carcinogenesis and progression, as well as a biomarker for the prediction of cancer risk and clinical prognosis.

## Abbreviations

SBP1: Selenium-binding protein 1; GPx1: Glutathione peroxidase 1; BE: Barrett’s esophagus

## Competing interests

Authors declare no conflict of interest.

## Authors’ contribution

WY and AD drafted the manuscript. Both authors read and approved the final manuscript.

## References

[B1] Group, KDR Epidemiologic studies on the etiologic relationship of selenium and Keshan disease Chin Med J (Engl) 1979 92 477 482 114372

[B2] Bellinger FP Raman AV Reeves MA Berry MJ Regulation and function of selenoproteins in human disease Biochem J 2009 422 11 22 10.1042/BJ20090219 19627257 PMC2912286

[B3] Greenwald P Anderson D Nelson SA Taylor PR Clinical trials of vitamin and mineral supplements for cancer prevention Am J Clin Nutr 2007 85 314S 317S 17209217 10.1093/ajcn/85.1.314S

[B4] Behne D Kyriakopoulos A Mammalian selenium-containing proteins Annu Rev Nutr 2001 21 453 473 10.1146/annurev.nutr.21.1.453 11375445

[B5] Kryukov GV Castellano S Novoselov SV Lobanov AV Zehtab O Guigo R Gladyshev VN Characterization of mammalian selenoproteomes Science 2003 300 1439 1443 10.1126/science.1083516 12775843

[B6] Gladyshev VN Hatfield DL Analysis of selenocysteine-containing proteins Curr Protoc Protein Sci, Chapter 2001 3 38 10.1002/0471140864.ps0308s20 18429173

[B7] Berry MJ Banu L Chen YY Mandel SJ Kieffer JD Harney JW Larsen PR Recognition of UGA as a selenocysteine codon in type I deiodinase requires sequences in the 3' untranslated region Nature 1991 353 273 276 10.1038/353273a0 1832744

[B8] Driscoll DM Copeland PR Mechanism and regulation of selenoprotein synthesis Annu Rev Nutr 2003 23 17 40 10.1146/annurev.nutr.23.011702.073318 12524431

[B9] Hu Y Benya RV Carroll RE Diamond AM Allelic loss of the gene for the GPX1 selenium-containing protein is a common event in cancer J Nutr 2005 135 3021S 3024S 16317164 10.1093/jn/135.12.3021S

[B10] Bansal MP Oborn CJ Danielson KG Medina D Evidence for two selenium-binding proteins distinct from glutathione peroxidase in mouse liver Carcinogenesis 1989 10 541 546 10.1093/carcin/10.3.541 2924398

[B11] Chang PW Tsui SK Liew C Lee CC Waye MM Fung KP Isolation, characterization, and chromosomal mapping of a novel cDNA clone encoding human selenium binding protein J Cell Biochem 1997 64 217 224 10.1002/(SICI)1097-4644(199702)64:2<217::AID-JCB5>3.0.CO;2-# 9027582

[B12] Porat A Sagiv Y Elazar Z A 56-kDa Selenium-binding Protein Participates in Intra-Golgi Protein Transport J Biol Chem 2000 275 14457 14465 10.1074/jbc.275.19.14457 10799528

[B13] Chen G Wang H Miller CT Thomas DG Gharib TG Misek DE Giordano TJ Orringer MB Hanash SM Beer DG Reduced selenium-binding protein 1 expression is associated with poor outcome in lung adenocarcinomas J Pathol 2004 202 321 329 10.1002/path.1524 14991897

[B14] Li T Yang W Li M Byun DS Tong C Nasser S Zhuang M Arango D Mariadason JM Augenlicht LH Expression of selenium-binding protein 1 characterizes intestinal cell maturation and predicts survival for patients with colorectal cancer Mol Nutr Food Res 2008 52 1289 1299 10.1002/mnfr.200700331 18435490

[B15] Scortegagna M Martin RJ Kladney RD Neumann RG Arbeit JM Hypoxia-inducible factor-1{alpha} suppresses squamous carcinogenic progression and epithelial-mesenchymal transition Cancer Res 2009 69 2638 2646 10.1158/0008-5472.CAN-08-3643 19276359 PMC2756430

[B16] Jeong J-Y Wang Y Sytkowski AJ Human selenium binding protein-1 (hSP56) interacts with VDU1 in a selenium-dependent manner Biochem Biophys Res Commun 2009 379 583 588 10.1016/j.bbrc.2008.12.110 19118533

[B17] Pass HI Liu Z Wali A Bueno R Land S Lott D Siddiq F Lonardo F Carbone M Draghici S Gene expression profiles predict survival and progression of pleural mesothelioma Clin Cancer Res 2004 10 849 859 10.1158/1078-0432.CCR-0607-3 14871960

[B18] Huang KC Park DC Ng SK Lee JY Ni X Ng WC Bandera CA Welch WR Berkowitz RS Mok SC Ng SW Selenium binding protein 1 in ovarian cancer Int J Cancer 2006 118 2433 2440 10.1002/ijc.21671 16380993

[B19] Kim H Kang HJ You KT Kim SH Lee KY Kim TI Kim C Song SY Kim HJ Lee C Kim H Suppression of human selenium-binding protein 1 is a late event in colorectal carcinogenesis and is associated with poor survival Proteomics 2006 6 3466 3476 10.1002/pmic.200500629 16645984

[B20] Pohl NM Tong C Fang W Bi X Li T Yang W Transcriptional regulation and biological functions of selenium-binding protein 1 in colorectal cancer in vitro and in nude mouse xenografts PLoS One 2009 4 e7774 10.1371/journal.pone.0007774 19924303 PMC2774949

[B21] Silvers AL Lin L Bass AJ Chen G Wang Z Thomas DG Lin J Giordano TJ Orringer MB Beer DG Chang AC Decreased selenium-binding protein 1 in esophageal adenocarcinoma results from posttranscriptional and epigenetic regulation and affects chemosensitivity Clin Cancer Res 2010 16 2009 2021 10.1158/1078-0432.CCR-09-2801 20332323 PMC2953959

[B22] Xia YJ Ma YY He XJ Wang HJ Ye ZY Tao HQ Suppression of selenium-binding protein 1 in gastric cancer is associated with poor survival Hum Pathol 2011 42 1620 1628 10.1016/j.humpath.2011.01.008 21497372

[B23] Zhang J Dong WG Lin J Reduced selenium-binding protein 1 is associated with poor survival rate in gastric carcinoma Med Oncol 2011 28 481 487 10.1007/s12032-010-9482-7 20354826

[B24] Zhang J Zhan N Dong WG Altered expression of selenium-binding protein 1 in gastric carcinoma and precursor lesions Med Oncol 2010 28 951 957 20480265 10.1007/s12032-010-9564-6

[B25] Zhang P Zhang C Wang X Liu F Sung CJ Quddus MR Lawrence WD The expression of selenium-binding protein 1 is decreased in uterine leiomyoma Diagn Pathol 2010 5 80 10.1186/1746-1596-5-80 21143902 PMC3014888

[B26] Huang C Ding G Gu C Zhou J Kuang M Ji Y He Y Kondo T Fan J Decreased selenium-binding protein 1 enhances glutathione peroxidase 1 activity and downregulates HIF-1alpha to promote hepatocellular carcinoma invasiveness Clin Cancer Res 2012 18 3042 3053 10.1158/1078-0432.CCR-12-0183 22512980

[B27] Stasio DI Volpe MG Colonna G Nazzaro M Polimeno M Scala S Castello G Costantini S A possible predictive marker of progression for hepatocellular carcinoma Oncol Lett 2012 2 1247 1251 10.3892/ol.2011.378 PMC3406508 22848296

[B28] Lamba G Ambrale S Lee B Gupta R Rafiyath SM Liu D Recent advances and novel agents for gastrointestinal stromal tumor (GIST) J Hematol Oncol 2012 5 21 10.1186/1756-8722-5-21 22569033 PMC3405472

[B29] Lamba G Gupta R Lee R Ambrale S Liu D Current management and prognostic features for gastrointestinal stromal tumor (GIST) Experimental Hematology & Oncology 2012 1 14 10.1186/2162-3619-1-14 23210689 PMC3514103

[B30] Fang W Goldberg ML Pohl NM Bi X Tong C Xiong B Koh TJ Diamond AM Yang W Functional and physical interaction between the selenium-binding protein 1 (SBP1) and the glutathione peroxidase 1 selenoprotein Carcinogenesis 2010 31 1360 1366 10.1093/carcin/bgq114 20530237 PMC2915633

[B31] Jerome-Morais A Wright ME Liu R Yang W Jackson MI Combs GF Jr Diamond AM Inverse association between glutathione peroxidase activity and both selenium-binding protein 1 levels and gleason score in human prostate tissue Prostate 2012 72 1006 1012 10.1002/pros.21506 22072582 PMC3288333

